# Antichlamydial Antibodies, Human Fertility, and Pregnancy Wastage

**DOI:** 10.1155/2011/525182

**Published:** 2011-09-22

**Authors:** Amanda J. Stephens, Mira Aubuchon, Danny J. Schust

**Affiliations:** Department of Obstetrics, Gynecology and Women's Health, University of Missouri School of Medicine, 500 North Keene Street, Suite 203, Columbia, MO 65201, USA

## Abstract

Genital infections with *Chlamydia trachomatis (C. trachomatis)* continue to be a worldwide epidemic. Immune response to chlamydia is important to both clearance of the disease and disease pathogenesis. Interindividual responses and current chlamydial control programs will have enormous effects on this disease and its control strategies. Humoral immune response to *C. trachomatis* occurs in humans and persistent antibody levels appear to be most directly correlated with more severe and longstanding disease and with reinfection. There is a close correlation between the presence of antichlamydial antibodies in females and tubal factor infertility; the closest associations have been found for antibodies against chlamydial heat shock proteins. The latter antibodies have also been shown to be useful among infertile patients with prior ectopic pregnancy, and their presence has been correlated with poor IVF outcomes, including early pregnancy loss. We review the existing literature on chlamydial antibody testing in infertile patients and present an algorithm for such testing in the infertile couple.

## 1. Introduction

*Chlamydia trachomatis (C. trachomatis)* infection is one of the most prevalent sexually transmitted diseases in the world. There were 409.2 cases per 100,000 population reported in the United States in 2009 [1].* C. trachomatis *is a common cause of urethritis, epididymitis, prostatitis, cervicitis, pelvic inflammatory disease, ectopic pregnancy, and tubal factor infertility (TFI). As many as 80% of cases are asymptomatic, particularly among females. This leads to continued transmission of the infection to sexual partners and the opportunity for chronic infection. 

Pelvic inflammatory disease (PID), an ascending infection from the cervix to the peritoneal cavity, is diagnosed in more than 800,000 women annually in the United States [2]. The most widely accepted microbial etiologies of PID are *Neisseria gonorrhoeae* and *C. trachomatis* [2–4]; still, other pathogens have been implicated and the final disease is almost certainly polymicrobial. While *C. trachomatis* infection may be a causative factor in up to 40% of cases of PID [5], fairly few women with *C. trachomatis* in the lower genital tract will progress to frank PID. The occurrence of symptomatic PID after untreated *C. trachomatis* infections may vary by population and time of followup but ranges between less than 2 and 9.5% [6, 7]. Most infected women will spontaneously clear their infections, although such clearance may take well over a year after infection [6, 8]. Women who do not clear their infections may suffer ascending infection and expansion into the full PID syndrome. 

**Figure 1:**
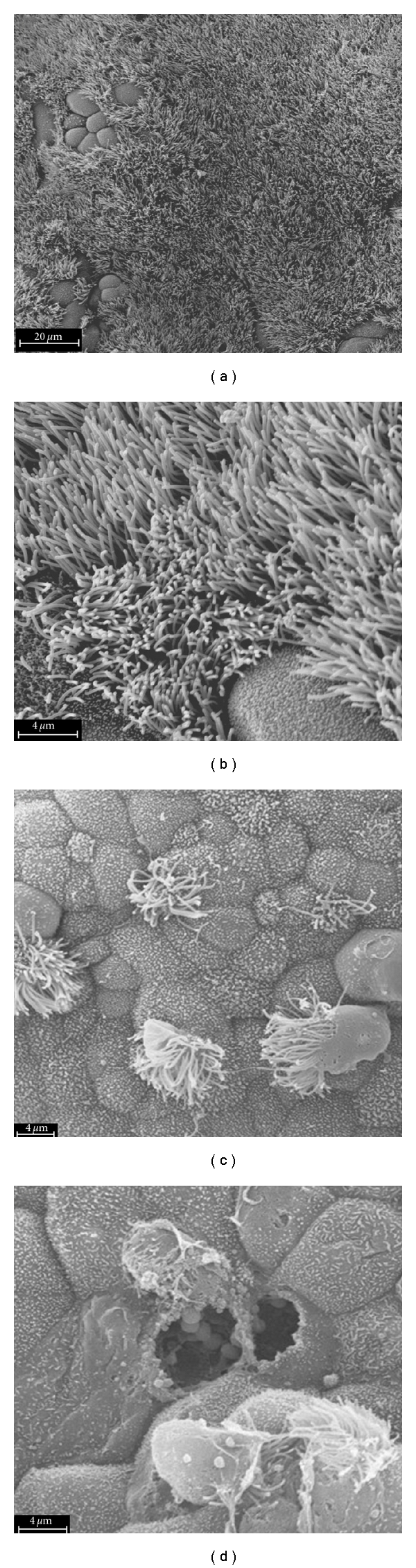
The effects of *C. trachomatis* infection on human fallopian tubal morphology. Human fallopian tubes in organ culture were left uninfected (a and b) or were infected with *C. trachomatis* serovar D (c and d). Ultrastructural analysis of the intratubal architecture uses scanning electron microscopy. Uninfected tubes are densely ciliated and contain intact secretory cells. The mucosal surface of *C. trachomatis*-infected tubes show remarkable deciliation and cellular disruption ([9], and reproduced with permission).

Once inflammation occurs in the fallopian tube, epithelial degeneration and deciliation of cells occur along the tube [2] (Figure 1 [9]). Edema in the fallopian tube exacerbates the intraluminal agglutination that occurs with endosalpingitis and leads to clubbing of the fimbriae and partial or complete tubal obstruction. Peritonitis caused by *C. trachomatis* can cause fibrinous exudates on the serosal surface of the uterus, fallopian tubes, and ovaries that fuses those structures to themselves and to surrounding bowel and omentum [2]. These adhesions are frequently associated with chronic pelvic pain. Each subsequent episode of PID doubles the risk for tubal factor infertility. Tubal pathology accounts for approximately 14% of subfertility [10]. Most women with tubal occlusion have no known history of sexually transmitted infections. Evaluation of tubal infertility may include serologic studies, hysterosalpingography, and laparoscopy. Intrauterine dye infusion during laparoscopy is the gold standard for assessing tubal occlusion, endometriosis, or pelvic adhesions in infertility patients. Laparoscopy, however, is a costly invasive test that has risk for complications. Hysterosalpingogram (HSG), a less costly and less complicated imaging modality, has a sensitivity of 65–96% and specificity of 73–83% for detecting tubal pathology [10–12]. This paper aims to evaluate the serologic tests available for *C. trachomatis* and their associations with TFI.

## 2. Pathogenesis of Disease

**Figure 2:**
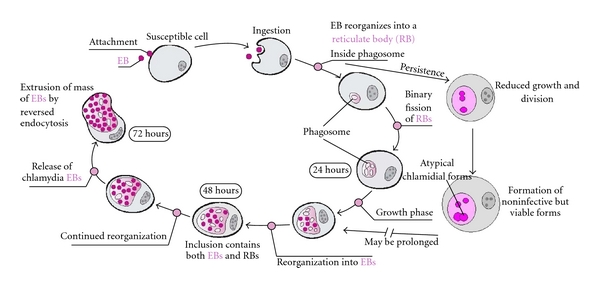
The life cycle of genital serovars of *C. trachomatis.* The chlamydial growth cycle involves transformation between distinct forms: the elementary body (EB) and the reticulate body (RB). The highly infectious EB attaches to nonciliated columnar or cuboidal epithelial cells and induces ingestion by the host cell. EB are metabolically inactive and represent the extracellular *C. trachomatis* growth form. Once ingested into a phagosome, fusion of the phagosome with the host lysosome is prevented, a highly unusual occurrence that ensures EB survival. The EB reorganizes within the phagosome into a metabolically active RB. RBs are noninfectious but can replicate and do so by binary fission. Several stimuli, including antibiotic and IFN*γ* exposure, can drive chlamydia into a persistent state, which lasts in vitro until removal of the exogenous stressor. If persistence is avoided, or if infection is reactivated from persistence, the RB will ultimately reorganize back into EB, which will be released from the host cell to infect surrounding epithelial cells (reproduced with permission [17]).

*C. trachomatis* is an obligate intracellular bacterium that produces a wide variety of clinical pathologies. Serovars D through K are pathologic to mucosal epithelial cells of the urogenital tract [13]. Erythema, edema, and mucopurulent discharge can be seen on physical exam during acute infection [14]. Urethritis, epididymitis, prostatitis, cervicitis, and pelvic inflammatory disease can develop following infection. With chronic infection, cellular changes including fibrosis and mononuclear cell infiltration lead to increased risk for ectopic pregnancy and TFI [14]. Both persistent infection and re-infection with *C. trachomatis* may be associated with worsening long-term sequelae, although the former appears to be the most consequential [15]. The ability of *C. trachomatis * to transform repeatedly from the resting form (elementary body; EB) to the replicative form (reticulate body; RB) enhances survival of the organism in the reproductive tract [16] (Figure 2 [17]). The EB of *C. trachomatis* attaches to the epithelial cell surface and incorporates into phagosomes that migrate to the distal region of the Golgi complex [13]. Lysosome fusion is prevented, and chlamydial infection averts immediate destruction. The EB then differentiates into the noninfectious but replicative reticulate body (RB) which further divides by binary fission [13]. Although *C. trachomatis* can partially evade immune detection [18–24] making infections fairly asymptomatic in many women, the infectious particles can be recognized by the host, with subsequent activation of host interferon (IFN-)  *γ* and proinflammatory cytokine secretion [25, 26]. In response to interferon exposure in vitro, RB can enter a persistent and noninflammatory state [26, 27]. Persistence can also be driven by nonsterilizing antibiotic exposure in vitro [14, 25–27]. Although there remains no direct in vivo evidence for persistence in humans, clinical scenarios suggest that persistent infection may remain undetected for many years and reactivation may occur much later in life. Reactivation may sometimes occur in women with prior infections who are now no longer sexually active or in women who have had their fallopian tubes electively obstructed and no longer have a patent route from lower to upper genital tract [14, 25, 28]. In the persistent state, chlamydial heat shock protein 60 (CHSP60) genes are upregulated and released [13, 29, 30]. In humans, elevated antibody responses to CHSP60 have been strongly associated with PID, ectopic pregnancy, scarring trachoma, and tubal infertility [13, 25, 29, 30]. Long-term exposure to CHSP60 may lead to a loss of tolerance to cross-reactive endogenous human antigens and generation of immune responses to conserved amino acid sequences that are also expressed in homologous human hsp60 [13, 25, 29, 30]. This immunity can lead to immune responses against human hsp60 in the early embryo and potentially link *C. trachomatis* infections to spontaneous pregnancy loss (see the following).

It remains unclear why some women clear their infections, while others endure long-lasting, ascending, and possibly persistent infections. The human cytokine IFN-*γ* may play a central role in this enigma. IFN-*γ* secretion by infected cells and by those cells brought in to control infection is central to infection clearance [6, 31]. IFN-*γ* is also involved in the tissue damage associated with *C. trachomatis* infections [6, 31]. Finally, in vitro models use low to moderate level IFN-*γ* exposure to drive *C. trachomatis* persistence with reactivation of the developmental cycle occurring after removal of the exogenous IFN [27]. The level of immune response to *C. trachomatis* may be affected by many factors. A large initial inoculation of infectious organisms may tip the balance toward exuberant responses that may clear the infection but be associated with more extensive damage. Genetic differences among infected subjects may alter response to infection. Several investigators have now reported on the effects of single nucleotide polymorphisms (SNPs) in inflammatory mediators, including cytokines, on the susceptibility to *C. trachomatis* infection and on the severity of tubal damage incurred during such infections [32, 33]. The level of oxygen within the fallopian tubes of *C. trachomatis* infected women may alter the balance of IFN-mediated clearance versus damage [34]. Reinfection may drive adverse infection sequelae among women who have already developed amnestic responses to *C. trachomatis.* Although some women spontaneously lose antibody responses to chlamydial antigens [35], antichlamydial IgG antibodies frequently persist for prolonged periods of time, even among women who have been treated with antibiotics [36, 37]. 

### 2.1. *C. trachomatis* Antibodies and TFI

Several immunologic techniques have been employed to study the relationship between the results of *C. trachomatis *serologic testing and the severity of *C. trachomatis*-associated pathologies, including TFI (summarized in Table 1). The most commonly studied antibodies include those directed against chlamydial IgG and CHSP60. The results of each of these investigations must be interpreted with caution, as the methodologies used to detect antibodies vary in their utility and the populations studied may vary in their genetic predisposition to immune responsivity and antibody production and persistence. Still the results appear to trend similarly. Elevated antichlamydial antibody levels can be found in >70% of women with tubal occlusion [12]. Malik et al. [38] evaluated IgG antibodies to *C. trachomatis* using enzyme-linked immunosorbent assays (ELISAs) and found that 63.6% of those with positive IgG serologies had tubal occlusion on HSG. This results in a sensitivity of 72.7% and specificity of 44.4% for tubal occlusion [38]. Perquin et al. [10] evaluated the presence of antichlamydial IgG antibodies using species-specific enzyme immunoassay (EIA) and compared these levels to findings of HSG and at laparoscopy. The sensitivity and specificity for IgG antibodies using EIA in predicting tubal pathology were found to be 45% and 83%, respectively [10]. Akande et al. [39] evaluated antichlamydial IgG antibodies using single-antigen inclusion tests and indirect immunofluorescence (whole-cell inclusion immunofluorescence; WIF). Antibody titers were found to be significantly higher among infertile women who had previously conceived compared to those with primary infertility [39]. This seemingly contradictory finding may be related to increased risk factors for sexually transmitted infections, including increased numbers of sexual partners, in those with secondary infertility, or with higher prevalence of other causes of infertility (e.g., anovulation or endometriosis) in those with primary infertility. Titers were significantly higher among those with a history of PID and those with documented tubal pathology. A linear relationship was observed between antibody titer level and the likelihood of tubal damage. Patients with the highest titers (>1 : 4096) had a 100% rate of tubal damage, and 73.1% of those had severe tubal damage [39]. Negative antibody testing did not preclude the diagnosis of tubal damage. den Hartog et al. [40] evaluated subfertile women with chlamydial antibody testing (CAT) involving antichlamydial Ig ELISA and high-sensitivity C-reactive protein (hsCRP) ELISA and compared the results to tubal evaluations using HSG. Seropositivity for IgG antibodies reflects previous *C. trachomatis* infection, while the presence of hsCRP reflects persistence of the infection. They found that the addition of HSG was of limited value in predicting tubal pathology in women who were CAT positive or CAT and hsCRP positive. 

Rodgers et al. [12] reported that TFI patients had higher titer levels of antichlamydial antibodies than either women in infertile control couples without TFI or in fertile control couples [12]. They also found that antibodies against ClpP were significantly higher in the TFI group compared to controls. ClpP is a proteolytic subunit of the ATP-dependent Clp protease complex that is part of a highly conserved serine protease family in eukaryotes and bacteria [12]. Chlamydial ClpP displays a 45% amino acid sequence identity with its homolog in humans. It is hypothesized that human antichlamydial ClpP antibodies can recognize cross-reactive epitopes and attack human ClpP in tissues [12]. 

Heat shock proteins (HSPs) are stress response proteins found in humans, animals, and bacteria. Expression of HSPs increases with temperature changes, ischemia, or hypoxia [12, 29, 30]. They have also been implicated in cell transformation and the development of metastatic potential and multidrug resistance [41]. HSPs contain amino acid regions that are highly conserved among the organisms that express them. Antibodies to CHSP60 have been linked to TFI in numerous studies [12, 25, 29, 30, 41]. CHSP60 stimulates inflammatory responses, including activation of macrophages and epithelial cells to secrete the inflammatory cytokines, IFN-*γ* and tumor necrosis factor-*α* (TNF-*α*), as well as other proinflammatory mediators [12, 41]. Stimulation of endothelial cells, smooth muscle cells, and macrophages leads to the production of adhesion factors and proinflammatory cytokines by activation of nuclear factor-*κ*B (NF-*κ*B) [41]. Jakus et al. [42] reported on the presence of anti-CHSP60 IgG antibodies in the follicular fluid of patients who had previously undergone IVF. Anti-CHSP60 antibodies were detected in 74.1% of women with implantation failure; 47.9% of those with 1–3 implantations per IVF cycle were anti-CHSP60 positive. Among women with documented tubal occlusion, 69.5% were antibody positive, while only 49.7% of women with other causes of infertility had anti-CHSP60 antibodies [42]. In another study, the presence of anti-HSP60 antibodies predicted TFI with a sensitivity of 56% but a specificity of 95%. Inclusion of anti-ClpP antibody testing increased the sensitivity to 69% [12]. The negative predictive value of ClpP and HSP60 for TFI was 79% and positive predictive value 92% [43]. Keltz et al. [11] assessed the sensitivity and specificity of HSG alone and HSG combined with CAT to detect tubal occlusion when compared to laparoscopy. HSG alone had sensitivity of 78% and specificity of 82%, while CAT alone had excellent positive predictive value of 94.8% but a poor negative predictive value of 69.8% due to its low sensitivity of 74%. This sensitivity increased to 97.3% when CAT results were combined with those of HSG. Laparoscopy, however, found pelvic pathologies other than tubal occlusion in 33–68% of patients with a normal HSG [11]. 

El Hakim et al. [44] evaluated 408 women with documented tubal damage on laparoscopy and compared the severity of damage with antibody titer levels using WIF. Similar to the results of the Keltz study [11], the severity of tubal damage was found to correlate significantly with increasing serum antibody levels [11, 44]. Bipolar and distal tubal occlusion were found to have the highest antibody titers [44]. 

Van Tetering et al. [45] evaluated 711 women with known anovulation and compared their antichlamydial antibody titers using ELISA with tubal pathology diagnosed using either HSG or laparoscopy. CAT screening yielded a sensitivity and specificity for tubal damage of 20% and 89%, respectively. However, the prevalence of CAT positivity was <5%, suggesting limited value for CAT screening in infertile women with known etiologic factors, particularly anovulation [45].

### 2.2. *C. trachomatis* Antibodies and Male Factor Infertility

While women can develop PID after *C. trachomatis* infections, men can develop urethritis, epididymitis, orchitis, and proctitis. Epididymitis can lead to canalicular system damage and obstructive azoospermia, although such severe post-chlamydial outcomes are uncommon. *C. trachomatis *infections more commonly result in the generation of antisperm antibodies and changes in semen quality that diminish rather than prevent male fertility. Detection of anti-*C. trachomatis* IgA and IgG antibodies, but not anti-HSP60 IgG antibodies, in male serum has been associated with poor semen characteristics and pregnancy rates regardless of female partner antibody status [46, 47] (summarized in Table 1). In one investigation, the presence of serum antichlamydial IgA, alone or in combination with IgG, correlates with reduced concentration and progressive motility of spermatozoa, an increase in the number of dead spermatozoa, poor sperm morphology, and a higher prevalence of leukocytospermia [46]. Men with IgG alone or IgA alone decrease their chance of achieving pregnancy by a third; men with both serum antibodies decrease their chances by almost two-thirds [46]. Interestingly, these reductions were completely surmounted through the use of in vitro fertilization (IVF). Not all investigators have replicated these findings. Eggert-Kruse et al. [48] examined male serum and seminal plasma from subfertile couples for antichlamydial IgA, IgG, and IgM antibodies using chlamydial lipopolysaccharide- (LPS-) directed ELISA. Although the presence of IgA antibodies in seminal fluid was associated with antibody detection in the serum of the female partners, the findings did not correlate with reduced sperm count or motility or subsequent fertilizing capacity [48].

### 2.3. *C. trachomatis* Antibodies and Pregnancy Outcomes

Antibodies to *C. trachomatis* and CHSP60 are strongly associated with TFI. Individuals with known TFI commonly undergo in vitro fertilization to overcome their tubal pathology, but they may still be at risk for adverse obstetric outcomes such as spontaneous abortion or biochemical pregnancy [13]. Among 174 women with normal fallopian tubes at laparoscopy who were followed for 3 years, the presence of antichlamydial antibodies using immunoflourescence (38.5%) was not predictive of obstetric outcomes [49]. The risk appears to be higher in the presence of antibodies specific to CHSP60. Human hsp60 is expressed during early embryo development and normally does not trigger an immune response [13, 42]. Heat shock proteins of various species contain a highly conserved region of amino acids. Sensitization to this conserved region in CHSP60 can result in reactivation of previously tolerized HSP60-specific lymphocytes. This may, in turn, compromise fetal or maternal cell viability via the direct activity of anti-HSP60 antibodies and/or the accompanying proinflammatory response. In 1999, Witkin [50] reported that women with cervical antichlamydial and anti-CHSP60 IgA antibodies were less likely to have a live birth after in vitro fertilization than their counterparts who did not have these antibodies. Patients undergoing IVF do not require patent or normal fallopian tubes. The rate of very early pregnancy loss was 3 times greater among those women who were anti-CHSP60 positive. Further, incubation of embryos in media containing human sera positive for anti-human HSP60 antibodies inhibited embryo development [50]. Supporting these results, Jakus et al. [42] studied 253 IVF patients and demonstrated lower implantation rates among women with follicular fluid anti-CHSP60 antibodies when compared with antibody negative controls. No differences were detected in the number of oocytes collected or the percentage that fertilized. 

Among women less than 35 years of age with a history of ectopic pregnancy treated with salpingectomy, the presence of serum anti-CHSP60 antibodies predicted lower spontaneous conception rates and decreased term delivery rates [51]. The same study reported that circulating IgG antibodies to a conserved epitope of CHSP60 (amino acids 260–271) were associated with decreased spontaneous fertility, repeated ectopic pregnancy, and adverse subsequent pregnancy outcome, suggesting that damage to the remaining tube may have occurred prior to the salpingectomy [51]. Women without antibodies to CHSP60 260–271 were five times more likely to have documented intrauterine conceptions and term deliveries when compared to those with positive serologies [51]. Women with positive serologies might therefore consider IVF after a first ectopic pregnancy to improve conception and pregnancy outcomes.

### 2.4. Use of Chlamydial Antibody Screening in Couples with Infertility

**Figure 3:**
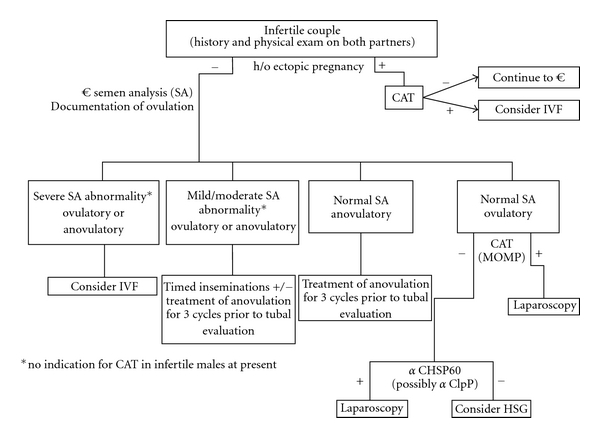
A proposed algorithm for use of chlamydial antibody screening in infertile couples.

Despite several continuing controversies, the existing data on the relationship between antichlamydial antibodies and tubal factor infertility make consideration of algorithms for screening possible (Figure 3). Chlamydial antibody screening is inexpensive when compared to other methods of tubal evaluation (HSG) but offers similar or improved sensitivity and specificity. HSG is more likely to be associated with adverse sequelae among women with chlamydial antibodies [40].

In our proposed algorithm, couples presenting with infertility would be screened first with careful histories and physical exams. If the female partner has a history of ectopic pregnancy, testing for anti-CHSP60 antibodies could be performed. Those with positive antibodies may be counseled to consider progression towards IVF to optimize their chance for a live birth. Couples without such a history would undergo documentation of ovulatory function and semen analysis. If the female was anovulatory, but the semen analysis was normal, the woman could consider ovulation induction and timed intercourse for 3-4 cycles prior to further interventions. If the woman was anovulatory or had normal ovulatory function, but the semen analysis revealed severe abnormalities, IVF would be recommended. If the semen analysis revealed persistent mild to moderate abnormalities, referral to an urologist could be considered and 3-4 cycles of timed intrauterine insemination undertaken prior to further evaluation. This would be combined with ovulation induction in anovulatory women. Couples with normal ovulation and normal semen parameters would be offered antichlamydial antibody testing (CAT). The significant body of literature linking CAT to TFI using anti- chlamydial major outer membrane proteins (MOMPs) antibodies warrants preliminary screening with standard CAT. Despite reports that CAT antibody levels can be associated with severity of tubal disease, the wide variations in antibody levels among affected patients reduce our enthusiasm for using antibody titer levels in management decisions at this time [44]. If CAT using MOMP is positive, laparoscopy would be recommended; if negative, testing for antibodies against CHSP60 (and possibly chlamydial Clp protease; ClpP) would be performed. Those positive for antichlamydial antibodies would proceed directly to laparoscopy for diagnosis and treatment. If normal fallopian tubes were found at laparoscopy, the couple could be treated using standard protocols for unexplained infertility [49]. Those negative for antibodies could also be treated for unexplained infertility. 

This suggested algorithm may be improved as additional antibody targets on chlamydia are identified and may need to be modified in light of current worldwide efforts to control the chlamydia epidemic [6, 52, 53]. Accompanying these control efforts are decreases in postinfection sequelae but continued increases in infection prevalence. It is possible that early identification and treatment of *C. trachomatis* infections is interrupting not only the destructive immune response to the pathogen but the protective response as well, a phenomenon that has been called “the arrested immunity hypothesis” [54]. Partner treatment may therefore be an important part of control paradigms as reinfection may become increasingly common. Does this arrested immunity result in reduced levels of protective IFN-*γ* that may promote persistence in some individuals? Arrested immunity might hinder antibody responses and make persistence of antibodies less common among previously infected but treated women. This may have little effect on screening programs if these women also avoid persistent infections. We do not presently have a definitive answer to these questions. Antibiotics such as penicillin and sulfonamides have been demonstrated to drive the development of chlamydial persistence in vitro [55]. Incomplete antibiotic therapy in areas that do not have availability of single-dose regimens might be predicted to promote persistence in vivo. Continued surveillance of chlamydial antibody screening programs for infertile couples will be key to monitoring the effects of control programs on the utility of screening. 

## 3. Conclusion

*C. trachomatis* is one of the most prevalent sexually transmitted infections in the United States and worldwide. Long-term sequelae of *C. trachomatis* infection include pelvic inflammatory disease, tubal factor infertility, and risk of ectopic pregnancy. Antibody testing for both antichlamydial IgG and anti-CHSP60 has been found to be associated with TFI. Increasingly high titers of antichlamydial IgG and anti-CHSP60 antibodies have been correlated with increasing severity of tubal damage when evaluated using HSG or laparoscopy. While sensitivity and specificity for CAT are comparable to that of HSG alone, CAT is less cost prohibitive and has less risks than either HSG or laparoscopy. CAT may be a valuable screening test prior to laparoscopy in infertility patients. Chlamydial antibodies have also been associated with male factor infertility, including reductions in sperm motility and total sperm counts. Chlamydial HSP60 antibodies have also been shown cross-react with human HSP60. This may lead to immune destruction of the early embryo and pregnancy wastage among women previously infected with *C. trachomatis*. Chlamydia antibody testing is a low-risk screening modality with sensitivity and specificity comparable to HSG and should be considered in the initial infertility evaluation. Limitations of CAT include (1) an inability to identify women with noninfectious causes of TFI, such as endometriosis, previous pelvic surgeries, or peritonitis, requiring these women to proceed with HSG or laparoscopy; (2) the possibility of declining antibody titers over time; (3) the ability to detect high titers among some women with normal appearing fallopian tubes; (4) the probability that additional antibody targets will be defined that improve screening sensitivity and specificity; (5) the unknown effects of chlamydial control programs on the utility of antibody testing among infertile couples.

## Figures and Tables

**Table 1:** Role of antichlamydial antibody testing in male and female fertility.

	Method	Sens. (%)	Specif. (%)	PPV (%)	NPV (%)	Utility in females	Utility in males
CT IgG [22]	ELISA	72.7	77.7	—	—	Presence indicates previous or persistent *C. trachomatis* infection; associated with tubal damage; increased titers associated with more severe tubal damage; sens./specif. may be increased with the addition of HSG or laparoscopy	—
CT IgG [33]	ELISA	43.2	86.5	63.3	73.8		—
CT IgG [6]	MIF	74	93	94.8	69.8		—
CT IgG [5]	EIA	45	83	—	—		—
CT IgG [23] Titer > 1 : 256	WIF	69	85	78	78		—
CT HSP60 [33]	ELISA	59.1	77.9	59.1	77.9	Reflects chronic *C. trachomatis* infection; predicts TFI	—
CT HSP60 [7]	GST ELISA	56	95	—	—	Higher titers related to increased severity of tubal damage	—
+ClpP Ab [7]	GST ELISA	69	—	92	79	Improve sens./specif. in Ab based diagnosis of TFI	—
CT IgA [28, 29]	MIF/EIA	—	—	—	—	—	Reduces chances of achieving pregnancy; reduced motility of spermatozoa, increased number of dead spermatozoa
+CT IgG [28, 29]	MIF/EIA	—	—	—	—	—	Further reduce pregnancy rates, decrease sperm concentration, decrease number of progressive spermatozoa
CT HSP60 [29]	ELISA	—	—	—	—	—	Reduce spermatozoa motility

ELISA: enzyme-linked immunosorbent assay, EIA: enzyme immunoassay, WIF: whole-cell inclusion immunofluorescence assay, GST ELISA: glutathione *S*-transferase ELISA, MIF: microimmunofluorescence, Ab: antibody; Sens.: sensitivity, Specif.: specificity, PPV: positive predictive value, NPV: negative predictive value; TFI: tubal factor infertility; HSG: hysterosalpingogram.
